# Effects of Acute Hypoxic Exposure in Simulated Altitude in Healthy Adults on Cognitive Performance: A Systematic Review and Meta-Analysis

**DOI:** 10.3390/biology13100835

**Published:** 2024-10-17

**Authors:** María Ramírez-delaCruz, Alfredo Bravo-Sánchez, Jorge Sánchez-Infante, Pablo Abián, Javier Abián-Vicén

**Affiliations:** 1 Performance and Sport Rehabilitation Laboratory, Faculty of Sports Sciences, University of Castilla-La Mancha, 45071 Toledo, Spain; maria.ramirez@uclm.es; 2 Faculty of Health Sciences, Universidad Francisco de Vitoria, Ctra. Pozuelo-Majadahonda km 1800, 28223 Pozuelo de Alarcón, Spain; alfredo.bravo@ufv.es (A.B.-S.); jorge.fisio.uclm@gmail.com (J.S.-I.); 3 Toledo Physiotherapy Research Group (GIFTO), Faculty of Physiotherapy and Nursing of Toledo, Universidad de Castilla-La Mancha, 45071 Toledo, Spain; 4 Faculty of Humanities and Social Sciences, Comillas Pontifical University, 28049 Madrid, Spain; pabian@comillas.edu

**Keywords:** hypoxia, reaction time, response accuracy, memory, attention

## Abstract

The neurocognitive response following hypoxia has received special interest. However, it is necessary to understand the impact of acute hypoxic exposure induced by simulated altitude on cognitive performance. This study aimed to determine the effects of acute hypoxic exposure in simulated altitude in healthy adults on reaction time, response accuracy, memory, and attention. Five electronic databases were searched. The inclusion criteria were: (1) Experimental studies involving a hypoxia intervention induced by a hypoxic air generator to determine the effects on cognitive performance; and (2) Conducted in adults (males and/or females; aged 18–50 years) without pathologies or health/mental problems. Four meta-analyses were performed: (1) reaction time, (2) response accuracy, (3) memory, and (4) attention. Finally, 37 studies were included in the meta-analysis. Hypoxia exposure induced detrimental effects on reaction time (standard mean difference (SMD) −0.23; 95% confidence interval (CI) −0.38–−0.07; *p* = 0.004), response accuracy (SMD −0.20; 95% CI −0.38–−0.03; *p* = 0.02), and memory (SMD −0.93; 95% CI: −1.68–−0.17; *p* = 0.02). Nevertheless, attention was not affected during hypoxia exposure (SMD −0.06; 95% CI: −0.23–0.11; *p* = 0.47). Acute exposure to hypoxia in controlled lab conditions appears to be detrimental to cognitive performance, specifically in reaction time, response accuracy, and memory.

## Simple Summary

Exposure to hypoxia may negatively affect cognitive performance. The aim of this systematic review and meta-analysis was to determine the effects of acute hypoxic exposure in simulated altitude in healthy adults on reaction time, response accuracy, memory, and attention. After a review and meta-analysis of the 37 studies analyzed, it appears that acute hypoxic exposure in simulated altitude produces impairment in reaction time, accuracy response, and memory in different cognitive tests in healthy adults. Nevertheless, attention shows no significant changes under hypoxic exposure in simulated altitude. Therefore, hypoxia training under controlled conditions could be a promising approach to avoid an impaired cognitive response in individuals who are frequently exposed to hypoxic environments.

## 1. Introduction

Oxygen is necessary for living organisms since it is used in several vital functions [1]. In humans, oxygen exchange occurs in the lung alveoli, where more than 95% of the oxygen diffuses into the blood. Then, this oxygen binds to hemoglobin and is transported to all organs of the body through the circulatory system [1,2]. The state of insufficient oxygen levels to maintain normal cellular function is defined as hypoxia [1], which can last for a short (i.e., acute) or long (i.e., chronic) period of time [2]. The most vulnerable organ to oxygen depletion is the brain due to its energy-supplied necessity, no glucose storage, and low capillary density [3]. Under hypoxic conditions, cerebral blood flow increases to maintain oxygen supply to the brain [4]. However, its compensatory mechanism due to reduced cerebral oxygen availability fails under severe hypoxia, lowering cerebral blood flow and increasing blood-brain barrier permeability [5]. In addition, oxygen deprivation deteriorates the development of interneuron connectivity and synaptic activity [6]. Therefore, hypoxia impairs neurological functions depending on its severity, which can lead to cognitive dysfunction.

Previous reviews about hypoxia exposure warn that it produces negative effects on cognition [7,8,9]. Cognitive impairment could be explained by the reduced oxygen saturation to the prefrontal cortex [10] since it is the primary brain region associated with greater activation during the performance of more complex central executive tasks [11]. Experimental studies have shown that cognitive performance is affected during expeditions at high altitudes (>3500 m) [12,13]. Furthermore, it was suggested that the greater the severity of hypoxia, the greater the cognitive deficit [14]. Despite the fact that these effects on cognitive performance may be only a temporary response [15], issues in reaction time, response accuracy, memory, and attention in individuals who are often exposed to those environments could produce potentially fatal consequences [16]. Nevertheless, these effects are controversial since other authors have not found alterations in cognitive qualities under hypoxic exposure (i.e., 4810 m [17]; 5260 m [18]; 7620 m [19]). Therefore, whether cognitive performance is impaired under hypoxia remains unclear.

These controversial results may be related to the great disparity in the methodologies of the studies. In addition to the heterogeneity of the cognitive tests, sample, and hypoxia exposure (i.e., duration and severity, altitude, and fraction of inspired oxygen (FiO_2_)), the controversy is probably explained mainly by the methods used to induce hypoxia (i.e., real or simulated altitude) [20]. The atmospheric partial pressure of oxygen decreases proportionally with the reduction of the barometric pressure at altitude, hypoxia induced at the laboratory does not. The physiological differences between normobaric and hypobaric hypoxia are currently in debate [21,22]. It is suggested that cerebral oxygenation and peripheral oxygen saturation are correlated with cognitive performance during simulated altitudes [20]. Although it has been reported that arterial oxygen saturation is lower during acute hypobaric exposures (i.e., real altitude environment) [23], the similar response in cerebral oxygenation after both conditions may translate to similar findings in cognitive performance. Nevertheless, technological progress nowadays leads us to clarify whether hypoxia conditions induced by simulated altitude (i.e., lab conditions using climate chambers and/or hypoxic generators) could produce detrimental effects on cognitive function. Therefore, the aim of this work was to determine the effects of acute hypoxic exposure in simulated altitude in healthy adults on the following cognitive performance variables: reaction time, response accuracy, memory, and attention. Due to the lack of clarity of cognitive terminology, memory analysis was centered on working and short-term memory responses. Similarly, attention and reaction time can often be confused throughout studies, and they were classified according to the intended cognitive variable analysis in the different studies.

## 2. Materials and Methods

This systematic review followed the preferred reporting items for systematic reviews and meta-analyses (PRISMA) [24] and was registered at the International Prospective Register of Systematic Reviews (PROSPERO; CRD42022348105).

### 2.1. Search Strategy and Study Selection

A systematic search was conducted for articles published up to 18th September 2023 describing the effects of hypoxia on cognitive performance. A manual search was performed in different electronic databases (PubMed, Scopus, Web of Science, MEDLINE, and SportDiscus) using a combination of these key terms: hypoxia, intervention, test, and cognitive performance. The search strategy used AND/OR operators in titles and abstracts. The complete search string is detailed in Appendix A. All articles found were collected and the duplicated studies were eliminated. Then, the titles and abstracts were independently reviewed (by two investigators: M.R.-d. and A.B.-S.) to identify articles that met the inclusion and exclusion criteria. If the two researchers could not agree, a third author (J.S.-I.) was consulted to make the final decision.

### 2.2. Eligibility Criteria

The selection of studies was based on the following inclusion criteria: (1) experimental studies involving a hypoxia intervention induced by a hypoxic air generator to determine the effects on cognitive performance and (2) conducted on healthy adults (i.e., males and/or females; aged 18–50 years; without pathologies or health/mental problems). We excluded articles meeting at least one of the following exclusion criteria: (1) were review articles, editorials, letters to the editor, or case reports; (2) were conducted in animals, cadavers, or in vitro; (3) did not provide data on normoxia/control or hypoxia conditions; (4) hypoxia was induced by altitude exposure; or (5) were observational studies that did not apply any type of hypoxia intervention.

### 2.3. Data Extraction

Data extraction was performed independently by two reviewers (M.R.-d. and P.A.). The full texts of each study were collected, and the necessary data were synthesized into a comprehensive table. Disagreements and discrepancies were resolved by a third author (A.B.-S.). In cases where essential data were missing in the text of the included studies, the authors were contacted to obtain the necessary information.

The following data were extracted: (1) name of the first author and year of publication; (2) sample size, age, and characteristics of the participants; (3) characteristics of the hypoxia intervention, where the percentage of FiO_2_ or altitude simulated in meters, duration, and hypoxia washout were collected; and (4) assessment of cognitive performance through different tests from which the following study variables were selected: reaction time, accuracy response, memory, and attention.

### 2.4. Methodological Quality Assessment

Before starting data extraction, a methodological quality assessment was performed using the Physiotherapy Evidence Database (PEDro) scale (Appendix B) [25]. The PEDro scale consists of 11 criteria that are scored with 1 point each if the criterion is correct. The total PEDro score ranges from 0 to 10 points, as criterion 1 is not included as part of the study quality rating because it pertains to external validity. Therefore, the quality assessment was interpreted using the following scale: 0–3 points were considered poor quality, 4–5 points were considered moderate quality, and 6–10 points were considered high quality [26].

The risk of bias assessment was used to evaluate the quality of the literature using Cochrane Robins 2.0 for randomized trials (Appendix C) [27]. The researchers performed the potential risk of bias assessment based on the following 7 items: (1) random sequence generation; (2) allocation concealment; (3) blinding of participants and personnel; (4) blinding of outcome assessment; (5) incomplete outcome data; (6) selective reporting; and (7) other biases. The overall assessment of the risk of bias was summarized as “low risk of bias,” “some concerns,” or “high risk of bias”.

Two researchers (M.R.-d. and A.B.-S.) independently performed the assessment of methodological quality (i.e., PEDro scale and Risk of Bias). In addition, the Kappa correlation test was used to analyze the level of agreement among authors to control for the risk of bias in the included studies (k = 0.91). Any discrepancies between the two investigators, such as disagreements on the scores in the quality assessment of the included studies, were judiciously resolved in a meeting by consensus with a third author (P.A.).

### 2.5. Statistical Analyses

Means ± standard deviation (SD) of outcomes under normoxia and hypoxia conditions were collected. Review Manager software (RevMan. Version 5.3. Copenhagen: Nordic Cochrane Centre, Cochrane Collaboration, 2014) was used for statistical analysis of the data. Four meta-analyses were performed: (1) reaction time; (2) accuracy response; (3) memory, and (4) attention. To assess heterogeneity between studies, the chi-square test and the Higgins I^2^ test were used [28]. The I^2^ ranges from 0% to 100%, where 0% indicates that no heterogeneity was observed, <25% indicates a low level, 25–75% indicates a moderate level, and >75% indicates a high level of heterogeneity [29,30]. Pooled odds ratios with 95% CI were calculated and a random-effects model using the Mantel–Haenszel method was used to pool the results of the different studies. The SMD and 95% CI were also used for the analysis of continuous data [31] and were interpreted as follows: trivial, <0.2; small effect, 0.2–0.5; moderate effect, 0.51–0.8; and large effect, >0.8 [32]. Statistical significance was set at *p* < 0.05.

## 3. Results

### 3.1. Search Results

**Figure 1:**
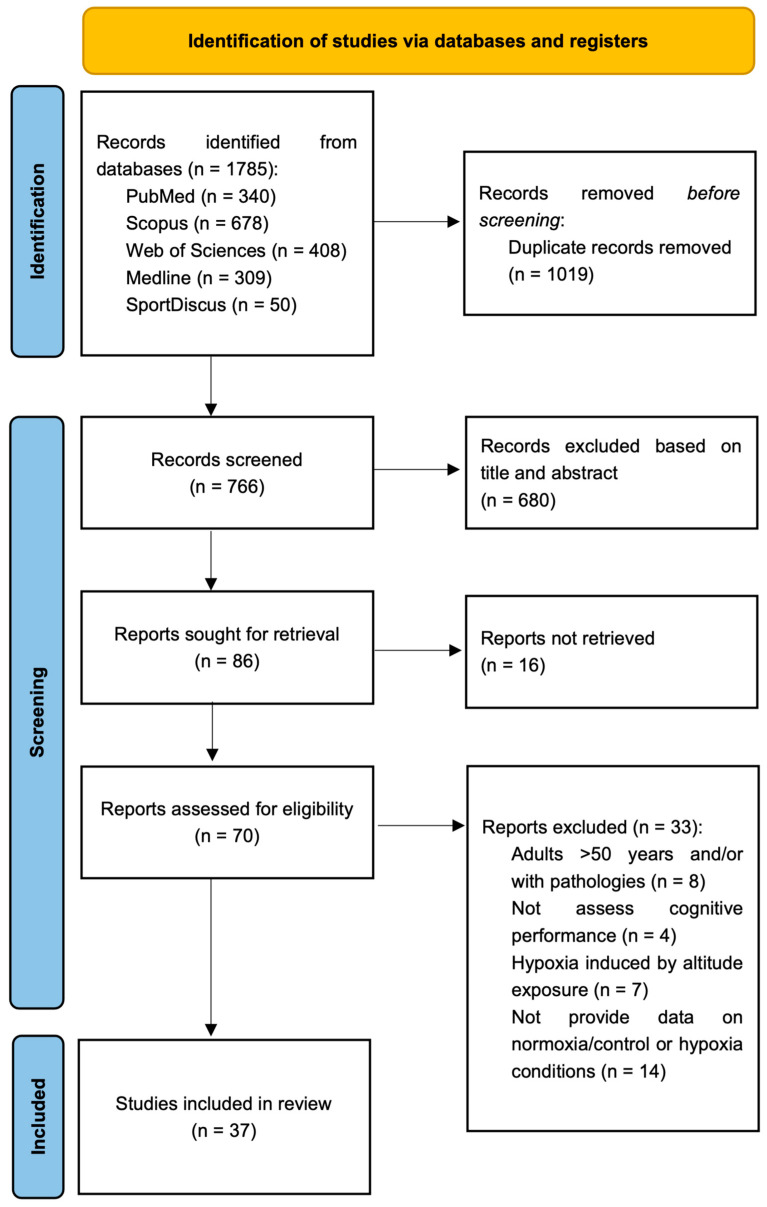
PRISMA flow diagram.

A total of 1785 articles were identified after the search in the selected electronic databases (PubMed: N = 340; Scopus: N = 678; Web of Science: N = 408; MEDLINE: N = 309; SportDiscus: N = 50). Initially, a total of 1019 duplicate studies were removed (EndNote X9, Clarivate Analytics). Then, the remaining 766 titles and abstracts were reviewed, and 86 studies were identified as suitable for further assessment. Following the evaluation of the full text of these 86 articles, 43 were excluded as not meeting the inclusion criteria. Since the data requested were not available in 5 studies, 37 articles were finally included in the meta-analysis (Figure 1).

The 37 articles included are depicted in Table 1. A total of 925 participants (543 males, 153 females, and 229 sex not defined) aged 18 to 45 were included. Hypoxia induced severity ranged from 1300 to 9500 m simulated altitudes or had a FiO_2_ of 18% to 6% (mean: 3526 m or FiO_2_ = 13.2%). The duration of hypoxic exposure ranged from 10 to 540 min (mean: 63.6 min). Of the studies included, 24 examined the effects of hypoxia on reaction time [10,33,34,35,36,37,38,39,40,41,42,43,44,45,46,47,48,49,50,51,52,53,54,55], 13 articles investigated the effects on response accuracy [10,33,38,39,40,43,44,45,51,53,56,57,58], 9 studies assessed the effects on memory [38,43,50,51,55,59,60,61,62], and 10 showed the effects on attention [43,50,57,61,63,64,65,66,67,68]. It should be noted that some of the 37 articles included cognitively performed responses during exercise intervention. However, for our analysis, only the data in hypoxia at rest were selected. In addition, some studies evaluated the same cognitive variable using different hypoxia doses and cognitive tests. These data were independently analyzed.

### 3.2. Assessment of Methodological Quality

**Figure 2:**
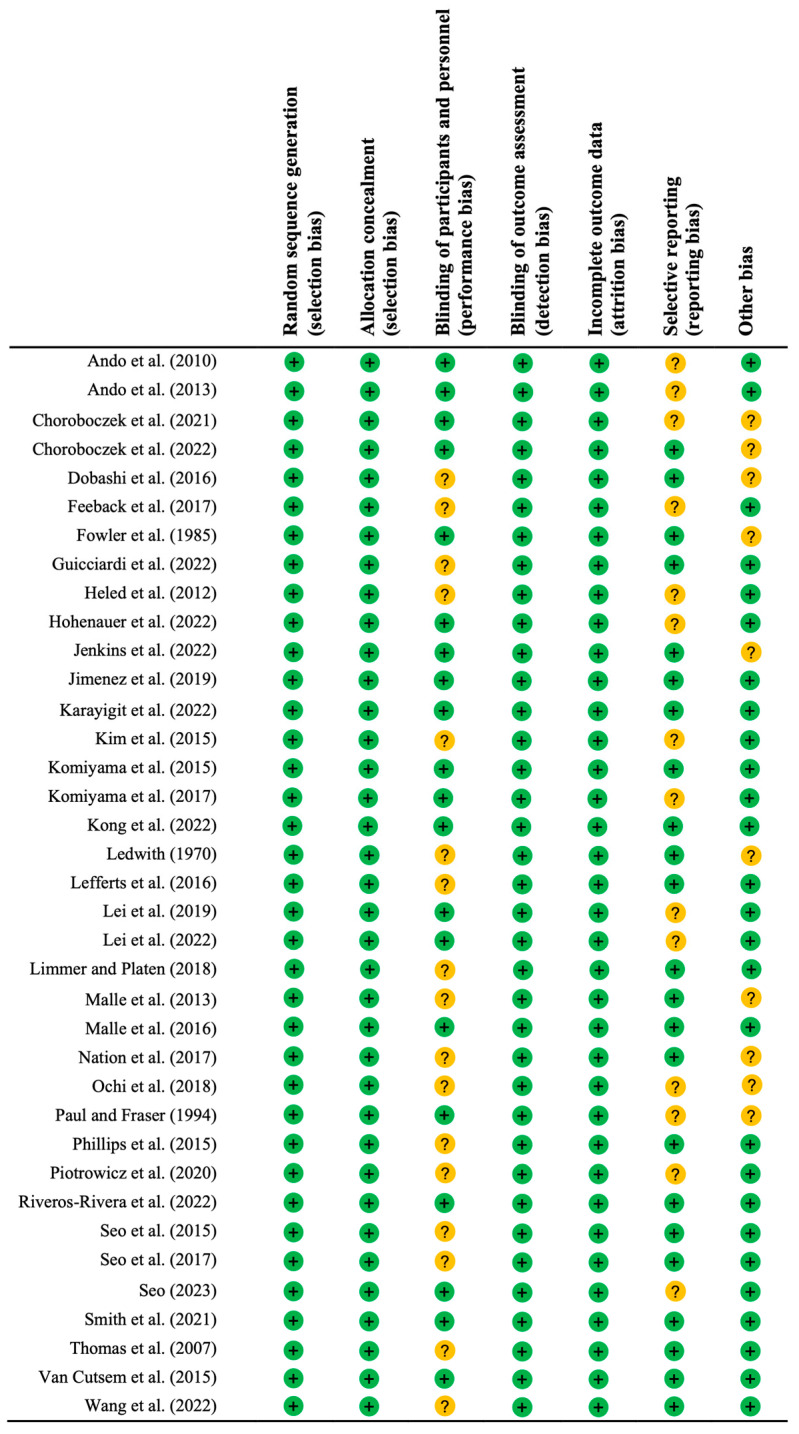
Overall assessment of risk of bias. Note: If a study’s scores are “+” in all subdomains, the overall rating is “low risk of bias”. When a study’s scores are “?” in one or more subdomains, the overall rating is considered “some concerns”. If a study’s scores are “-” in one or more subdomains, the overall rating is “high risk of bias”, giving rise to substantial doubts about the quality of the research [10,33,34,35,36,37,38,39,40,41,42,43,44,45,46,47,48,49,50,51,52,53,54,55,56,57,58,59,60,61,62,63,64,65,66,67,68].

**Figure 3:**
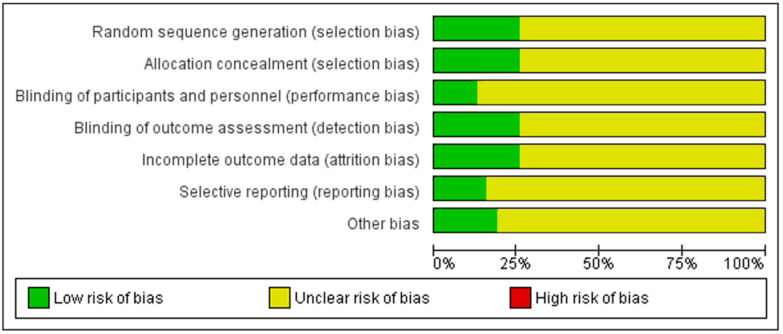
Risk of bias assessment of the included trials.

PEDro scale scores ranged from 4 to 9 (6.02 ± 1.40; Appendix B Table A1). Studies showed high methodological quality, due to the results provided (criterion 8 to 11) throughout randomized designs (criterion 2) and blinding of subjects (criterion 5). The risk of bias assessment (Figure 2 and Figure 3, Appendix C Table A2) showed “some concerns” in 29 of the 37 included studies, considering 8 studies as “low risk of bias”.

### 3.3. Meta-Analysis Results

**Figure 4:**
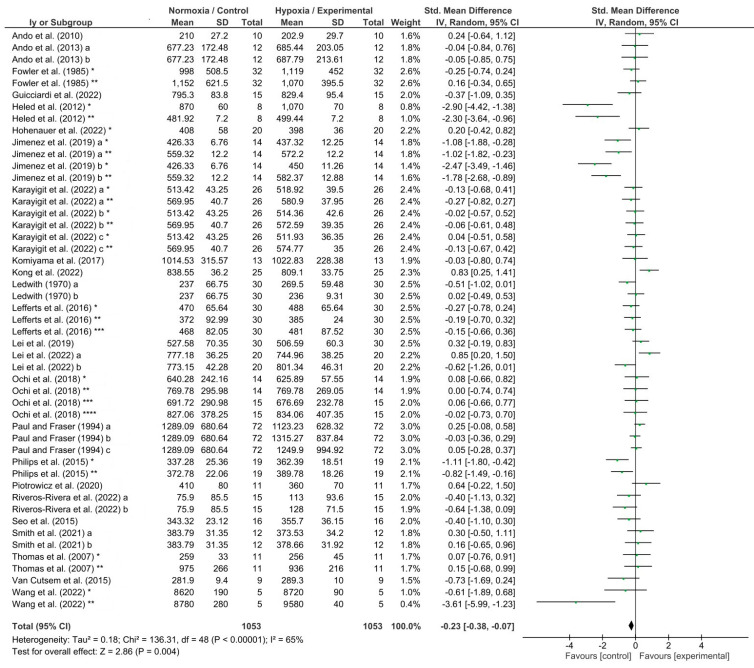
Forest plot of studies evaluating the effects of acute hypoxic exposure in simulated altitude on reaction time. a, b, and c, different hypoxia exposures; *, **, ***, and ****, different reaction time tests [10,33,34,35,36,37,38,39,40,41,42,43,44,45,46,47,48,49,50,51,52,53,54,55].

**Figure 5:**
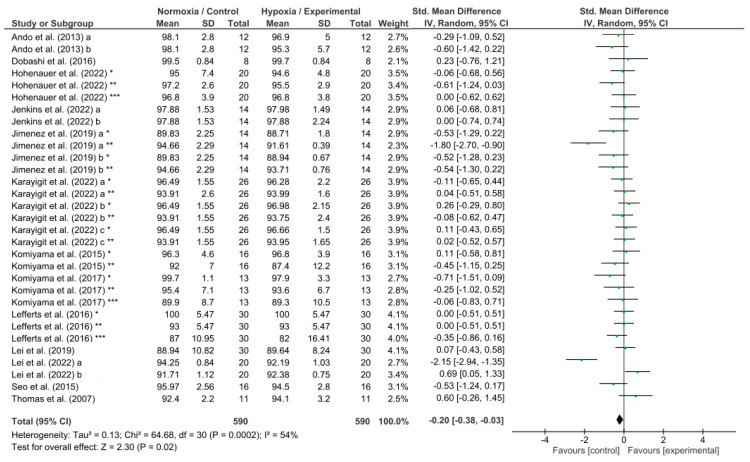
Forest plot of studies evaluating the effects of acute hypoxic exposure in simulated altitude on response accuracy. a, b, and c, different hypoxia exposures; *, **, and ***, different response accuracy tests [10,33,38,39,40,43,44,45,51,53,56,57,58].

**Figure 6:**
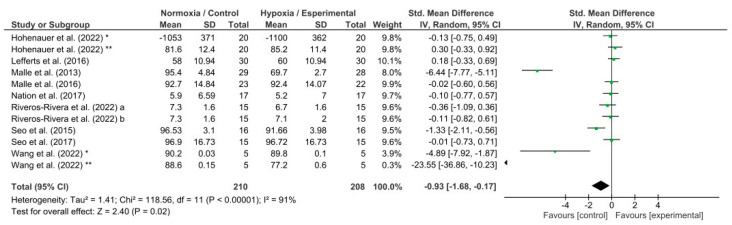
Forest plot of studies evaluating the effects of acute hypoxic exposure in simulated altitude on memory. a, and b, different hypoxia exposures; *, and **, different memory tests [38,43,50,51,55,59,60,62].

**Figure 7:**
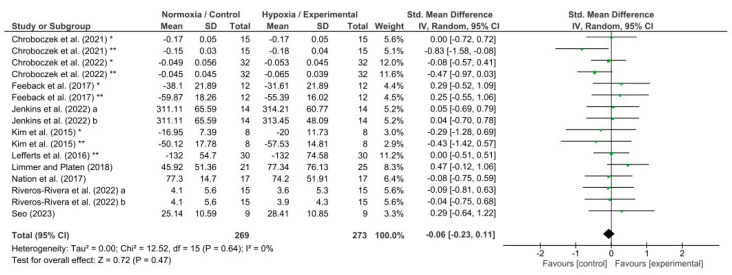
Forest plot of studies evaluating the effects of acute hypoxic exposure in simulated altitude on attention. a, and b, different hypoxia exposures; *, and **, different attention tests [46,50,57,63,64,65,66,67,68].

The effects of hypoxia conditions on reaction time, response accuracy, memory, and attention were evaluated by four different meta-analyses which showed low to high heterogeneity (reaction time, I^2^ = 65%; response accuracy, I^2^ = 54%; memory, I^2^ = 91%; and attention, I^2^ = 0%). Hypoxia conditions induced a detrimental effect on reaction time (*p* = 0.004; SMD −0.23; 95% CI −0.38–−0.07; Z = 2.86; Figure 4). In addition, hypoxia exposure showed a significant decrease in response accuracy (*p* = 0.02; SMD −0.20; 95% CI −0.38–−0.03; Z = 2.30; Figure 5) and memory (*p* = 0.02; SMD −0.93; 95% CI: −1.68–−0.17; Z = 2.40; Figure 6). Nevertheless, attention was not affected during hypoxia conditions (*p* = 0.47; SMD −0.06; 95% CI: −0.23–0.11; Z = 0.72; Figure 7).

## 4. Discussion

Exposure to hypoxia induced by altitude has a stated marked influence on cerebrovascular function and neurocognitive performance [69]. Nevertheless, the cognitive response under hypoxic conditions induced by simulated altitude is not clarified. In this systematic review, 37 studies were included in the meta-analysis. The principal findings of our work were that reaction time, response accuracy, and memory are negatively affected under hypoxic conditions; meanwhile, no changes in attention were observed. Therefore, our meta-analysis suggests that hypoxia exposure induced by simulated altitude (i.e., controlled lab conditions) has detrimental effects on cognitive performance.

### 4.1. Effects of Hypoxia on Reaction Time

Reaction time, which is defined as the time from the appearance of an unanticipated stimulus to the start of the response (motor activity) by the person [70], is one of the most important measures of human performance in many life situations [71]. Many studies and literature reviews have shown that exposure to hypoxia induced by altitude negatively affects cognitive performance, specifically reaction time [72,73,74]. McFarland in 1937, was one of the first to evidence an impaired psychomotor reaction time while individuals were at high altitudes in the Andes [75]. Our results from 24 studies included where reaction time assessment was carried out under simulated altitudes (i.e., 1300–5500 m; FiO_2_ = 18–10%), have shown a significantly detrimental effect on reaction time under hypoxia induced by a hypoxic generator (*p* < 0.004; Figure 4). The hypoxia-induced impaired reaction time is not surprising since reaction time is basically composed of a cognitive or “premotor” part (coding and actual decision process) and a motor part (response execution) [43], and the cognitive part is the cornerstone in the response [76]. Moreover, the motor part of the reaction time is assessed throughout the test, and the anaerobic system, which supplies energy during the quick motor response, is not affected by low O2 saturation levels [77]. Ando et al. (2010) [34] found that the premotor time to peripheral visual stimuli was significantly increased under hypoxia (2200 m; FIO_2_ = 16%) and was closely associated with a decrease in cerebral oxygenation. Cognitive function depends on a continuous supply of oxygen to the brain [78]. Under hypoxia, arterial O2 pressure and saturation are decreased which may compromise cerebral oxygenation [34,79,80]. Therefore, the compromised oxygen supply to the brain could alter cognitive function, causing a delayed reaction to the stimuli.

### 4.2. Effects of Hypoxia on Response Accuracy

Response accuracy is one of the most common measures of executive function [81]. It is used as a performance indicator for different cognitive tasks [82], being commonly calculated as the percentage of correct trials (correct trials divided by the total number of trials) of the participant [33,83]. It suggested that acute hypoxia exposure impairs central executive function, worsening response accuracy [10,33,39,51] in a saturation of peripheral O2 (SpO_2_) and cerebral oxygenation reduction-dependent manner [20]. Nevertheless, the effects of hypoxia exposure at altitude on response accuracy are less clear than the effects on reaction time, finding studies that show an impairment or no significant changes [73]. Our meta-analysis has found a negative effect on the response accuracy under hypoxia induced by simulated altitude (*p* = 0.02; Figure 5) when compared with the control/normoxia group. In the studies analyzed, the effects of the induced hypoxia have been evaluated under moderate to low FiO_2_ percentages (i.e., 11.2%–18%). It should be stated that the studies with lower FiO_2_ percentages (Lei et al. (2022) [45] = 11.2%; Karayigit et al. (2022) [40] and Lei et al. (2019) [44] = 12%; and Thomas et al. (2007) [53] = 13%), despite having oxygen saturations or even greater discomfort (altitude sickness), have shown better or similar response accuracy scores in the experimental/hypoxia group than the control/normoxia group. On the contrary, the negative effects are notably significant under higher FiO_2_ (~15%) [38,39,45]. It seems that participants aiming to avoid failure and maintain their response accuracy, respond slower to the stimuli affecting their reaction time as Steinman et al. (2023) [84] have found in their pilots who tried to make fewer errors by trading response speed for greater response accuracy. Therefore, the effects of hypoxia on response accuracy would be more noticeable when the reaction time is not negatively affected by reductions in SpO_2_ and cerebral oxygenation. Furthermore, future research should include effectiveness (accuracy) and efficiency (accuracy to time ratio) indices [81].

### 4.3. Effects of Hypoxia on Memory

Exposure to hypoxia can trigger unfavorable effects in the hippocampus–prefrontal cortex pathway, which is crucial in memory processing [85,86]. The hippocampus is where memories are stored and memory retrieval is facilitated [87,88]. The hippocampus is one of the brain structures most susceptible to oxygen deprivation [89]. Therefore, acute hypoxia can induce hippocampal damage, impaired hippocampus–prefrontal cortex synaptic plasticity, and thus, a cognitive impairment [90]. It suggested that a hypoxic environment induced by altitude impairs memory [12,91]. Our meta-analysis has already shown a significantly detrimental effect on memory under hypoxia generated by simulated altitude (*p* = 0.02; Figure 6). It seems that the effects on the prefrontal cortex can explain the impairment through memory tasks. Wang et al. (2022) [55] assessed the hemodynamic activity of the prefrontal cortex using a near-infrared spectroscopy system during participants were performing their memory tasks. They observed a reduced activation in the left hemisphere of the dorsolateral prefrontal cortex under hypoxia than normoxia conditions, leading to an impaired memory capacity. Therefore, the damage produced on the hippocampus and prefrontal cortex under hypoxia in both real and simulated altitude could explain the detrimental effects confirmed on the memory.

### 4.4. Effects of Hypoxia on Attention

Previous studies have not reached a definitive conclusion regarding the effect of hypoxia on attention [92], which recently has received special research interest in the neurophysiological field. Our findings have shown that there are no significant differences in attentional performance of the different attentional tests analyzed between hypoxia or normoxia exposures. While some research showed worse results on attentional tasks when people were subjected to hypoxic exposure [61,63,64,66], others found slightly improved attention test scores [65,67,68]. Attention is essential to focus on the information selected [92], but the managing process lead by the central nervous system is a complex network issue. The attention network has three functions (i.e., alerting, orienting, and executive control). Each function is associated with different brain regions [92]. Thus, despite it being suggested that acute hypoxia clearly impairs reaction time, response accuracy, and memory, its effects may differ for attention due to the complex network led by different brain regions [93]. Moreover, due to the limited literature, which is mainly contradictory, further research is needed to obtain conclusive results.

### 4.5. Harmful Effects of Hypoxia Exposure

It has been reported that between 2000 m and 4000 m altitude, the incidence and severity of acute mountain sickness (AMS) in non-acclimatized healthy populations can rapidly increase from 20% to 70% [94]. Moreover, it is well established that, under hypoxic conditions, AMS varies with duration: as the exposure time increases, the discomfort experienced increases [95]. Imray et al. (1998) [96] found a negative correlation between the degree of AMS and cerebral oxygenation. One of the most widely used tools to measure perceived symptoms of AMS considered detrimental to health is the self-reported Lake Louise Score (LLS) [97,98]. Some of the studies included in this systematic review evaluated AMS with contradictory results. In the study of Guicciardi et al. (2022) [36], no significant differences were found in the hypoxia versus normoxia condition. However, Hohenauer et al. (2022) [38], who evaluated AMS symptoms at the end of each experimental measurement, detected a significant difference in scores between the two conditions. On the other hand, Limmer and Platen (2018) [67], despite being among the few studies evaluating AMS, consider the use of LLS as a limitation of their study. Therefore, it has been suggested that the use of the self-reported LLS questionnaire leads to different assessments of AMS in hypobaric hypoxia compared to normobaric hypoxia [95]. Furthermore, although headache is considered a main symptom of AMS, West, (2011) [99] proposes that it should not be a mandatory symptom for the diagnosis of AMS. Others recommend assessing AMS only after 6 h to avoid confusing acute mountain sickness with other symptoms of confusion (e.g., travel, vagal response) [100]. However, despite these limitations, the self-reported LLS questionnaire is still highly recommended due to its simplicity and is the most popular questionnaire in current use [67,101].

### 4.6. Study Limitations

This systematic review and meta-analysis are not free of limitations. Cognitive function is related to different cognitive domains that activate different regions of the brain [102], being unclear which are most vulnerable to hypoxia [14,73]. Furthermore, the biological variability of individual physiological responses following exposure to hypoxia [103] and the possible cognitive test familiarization in the different studies analyzed could be a limitation of our meta-analysis. Lastly, studies included in our meta-analysis presented different hypoxic protocols (i.e., durations and severity) and methods used to evaluate reaction time, accuracy response, memory, and attention, so the heterogeneity of the added studies is high, and this should be considered as a limitation. Nevertheless, we have evaluated the acute effects following exposure to hypoxia induced in controlled lab conditions (with generator or chamber) on cognitive function tested by a validated assessment. Finally, further research is needed to be able to differentiate hypoxic responses during normobaric and hypobaric lab conditions.

## 5. Conclusions

This is one of the first systematic reviews and meta-analyses that provides an overview of published studies on the effects of acute hypoxia exposure induced by simulated altitude on cognitive performance (i.e., reaction time, response accuracy, memory, and attention) in healthy adults. Our results have shown that acute exposure to hypoxia in controlled lab conditions appears to be detrimental to cognitive performance, specifically in reaction time, response accuracy, and memory, although attention does not appear to be affected. Moreover, it seems that the impaired cognitive function is mainly explained by the compromised oxygen supply, which causes a delayed failed reaction to the stimuli and reduced activation of the different regions of the brain responsible for developing memory capacity. Future directions should be toward the effects of repeated bouts of hypoxia exposure (i.e., chronic exposure) on cognitive function adaptations.

## 6. Practical Application

These findings have important implications for individuals who are often exposed to those environments, such as the military (e.g., pilots, parachutists) and emergency service workers (e.g., firefighters, medical technicians, paramedics, mountain rescue) and even extreme sports athletes (e.g., climbers, alpinists, divers), since their impaired cognitive response presented could produce potentially fatal consequences [16]. Then, interventions are needed to reduce the detrimental effects of acute hypoxia on cognitive function in these individuals. Training in hypoxia under controlled conditions is a promising approach. In addition, the benefits of living and training under hypoxic conditions in improving the altitude performance of athletes due to neuromuscular and cardiovascular system adaptations are well known [104]. Nevertheless, the effects of training under hypoxia on a sport’s cognitive aspects are unknown, and further research is needed to understand one of the cornerstones of successful athletic performance.

## 7. Key Points

- Altitude causes detrimental effects on cognitive performance due to hypoxia; however, the response induced by simulated altitude was unknown.
- Acute hypoxic exposure in simulated altitude produces an impairment in reaction time, accuracy response, and memory on different cognitive tests in healthy adults.
- Nevertheless, attention shows no significant changes under hypoxic exposure in simulated altitude.

## Figures and Tables

**Table 1:** Summary of included studies.

Study	Population			Characteristics of Hypoxia Exposure	Selected Variables			
	Sample Size (N): Male (M)/Female (F)	Age (Years)	Characteristics		Reaction Time	Response Accuracy	Memory	Attention
Ando et al. (2010) [34]	N = 10 (M)	25.1 ± 3.4	Any history of cardiovascular, cerebrovascular, or respiratory disease	FiO_2_ 16% (2200 m)	RT measurement apparatus (Qtec, Osaka) (ms)			
Ando et al. (2013) [33]	N = 12 (M)	22.9 ± 1.5	No regular training, physically active, any history of cardiovascular, cerebrovascular, or respiratory disease	FiO_2_ 18% (1300 m) FiO_2_ 15% (2600 m)	Go/No Go Test (ms)	Go/No Go Test (%)		
Chroboczek et al. (2021) [63]	N = 15	23.1 ± 2.1	Healthy, non-obese young adults	30 min → FiO_2_ 13% (3500 m) 30 min → FiO_2_ 12% (4500 m) 30 min → FiO_2_ 11% (5500 m) Washout: 1 week				Stroop reading interference (s) Stroop naming interference (s)
Chroboczek et al. (2022) [64]	N = 32 (M)	20.4 ± 0.6	Physical Education and Sport students	30 min →FiO_2_ 13% (3500 m) Washout: 2 weeks				Stroop reading interference (s) Stroop naming interference (s)
Dobashi et al. (2016) [56]	N = 8	23.5 ± 2.2	People capable of high-intensity cycling. No history of cardiovascular, cerebrovascular, or respiratory disease	FiO_2_ 14.1% (3200 m)		Stroop Test (%)		
Feeback et al. (2017) [65]	N = 12 (M)	18 to 25	Healthy and non-smokers. African Americans (N = 6) and Caucasian (N = 6)	FiO_2_ 12% (4300 m)				TMT-A (s) TMT-B (s)
Fowler et al. (1985) [35]	N = 32	19 to 32	Students	FiO_2_ 11–16%	Mannikin Test (ms)			
Guicciardi et al. (2022) [36]	N = 15 (M)	30.2 ± 6.6	Athletes involved in regular endurance training for at least 3 years (8 h/week), without chronic cardiopulmonary, metabolic, or neurological disease	18 min → FiO_2_ 13%	The Bivalent Shape Task (ms)			
Heled et al. (2012) [37]	N = 8 (M)	23 ± 3	Healthy young people	10 min → FiO_2_ 15.6% (2400 m)	Visual Vigilance Task (ms) 4-Choice RT (ms)			
Hohenauer et al. (2022) [38]	N = 20 (10M/10F)	Males = 30.3 ± 6.3 Females = 24.8 ± 5.1	Healthy, non-smokers, recreationally trained, and free of any known cardiovascular, respiratory, or neurological disorders	15 min → FiO_2_ 14.4% (2980 m) Washout: 1 week	2-Choice RT (ms)	Mannikin Test (%) Switching Test (%) N-back (%)	N-back (ms) N-back (%)	
Jenkins et al. (2022) [57]	N = 14 (10M/4F)	Males = 27.6 ± 1.5 Females = 26.7 ± 1.3	Recreationally active people (8.54 ± 1.44 h/week of physical activity), without musculoskeletal, neurological, or cardiovascular disorders	60 min → FiO_2_ 16% (2133 m) 60 min → FiO_2_ 14.3% (3048 m) Washout: 48 h		Stroop Test (%)		Stroop Test (points)
Jimenez et al. (2019) [39]	N = 14 (9M/5F)	Males = 24.7 ± 3.6 Females = 27.6 ± 4.4	Recreationally active, right-handed individuals. No history of physical or mental health problems, no medication, and no neuroactive drugs	45 min → FiO_2_ 15.4%, (2400 m) 45 min → FiO_2_ 12.8% (3900 m) Washout: 48 h	Eriksen Flanker Test (ms) Stroop Test (ms)	Eriksen Flanker Test (%) Stroop Test (%)		
Karayigit et al. (2022) [40]	N = 26 (13M/13F)	Males = 23.6 ± 2.8 Females = 22.8 ± 1.4	Healthy, non-smokers. With at least three years of resistance training experience, and who trains four times per week (squats and bench presses)	40 min → FiO_2_ 16%, (2000 m) 40 min → FiO_2_ 14% (3000 m) 40 min → FiO_2_ 12% (4000 m) Washout: 72 h	Eriksen Flanker Test (ms)	Eriksen Flanker Test (%)		
Kim et al. (2015) [66]	N = 8 (M)	41.0 ± 2.0	Healthy, low-altitude residents who had not been exposed to normobaric hypoxia or altitudes above 2500 m in the previous 2 months	FiO_2_ 12.5% (4300 m)				TMT-A (s) TMT-B (s)
Komiyama et al. (2015) [58]	N = 16 (M)	23.0 ± 2.3	Physically active people with no history of cardiovascular, respiratory, or cerebrovascular diseases	10 min → FiO_2_ 15% (2600 m) Washout: non-consecutive sessions		Spatial Delayed Response Task (%) Go/No Go Test (%)		
Komiyama et al. (2017) [10]	N = 13 (M)	21.5 ± 3.5	Physically active people with no history of cardiovascular, respiratory, or cerebrovascular diseases	FiO_2_ 12–13% (4500 m–3800 m)	Go/No Go Test (ms)	Spatial Delayed Response Task (%) Go/No Go Test—Go Trial (%) Go/No Go Test—No Go Trial (%)		
Kong et al. (2022) [41]	N = 25 (M)	22.2 ± 2.4	Physically active men	30 min → FiO_2_ 11% (5000 m)	Stroop Test (ms)			
Ledwith (1970) [42]	N = 30 (24M/6F)	18 to 45	First-year psychology students (N = 19) or members of the St. John Ambulance Society (N = 11)	2133 m 4267 m	Choice RT (ms)			
Lefferts et al. (2016) [43]	N = 30 (15M/15F)	21.0 ± 4.0	Healthy recreationally active people	120 min → FiO_2_ 12.5% Washout: at least 24 h	Eriksen Flanker Test (ms) N-back (ms)	Eriksen Flanker Test (%) N-back (%)	N-back (%)	Eriksen Flanker Test (ms)
Lei et al. (2019) [44]	N = 30 (F)	22.6 ± 3.2	Healthy and sedentary young women	FiO_2_ 12% (4000 m) Washout: 72h	Go/No Go Test (ms)	Go/No Go Test (%)		
Lei et al. (2022) [45]	N = 20 (M)	21.4 ± 2.0	Recreationally active men	FiO_2_ 15.4% (2500 m) FiO_2_ 11.2% (5000 m) Washout: 3–7 days	Stroop Test (ms)	Stroop Test (%)		
Limmer and Platen (2018) [67]	N = 80 (51M/29F) HYP = 25 NOR = 21	Males = 25.5 ± 6.0 Females = 24.8 ± 5.9	Healthy young adults	FiO_2_ 10% (5800 m)				Learning Effect—(attentional performance value)
Malle et al. (2013) [60]	N = 57 (M) HYP = 28 NOR = 29	HYP = 23.9 ± 1.7 NOR = 23.9 ± 2.8	Healthy, non-smoking, right-handed male pilots.	Progressive ascent up to 9500 m and return to ground level (750 m/min)			Paced Auditory Serial Addition Test (%)	
Malle et al. (2016) [59]	N = 86 (M) NOR = 23 HYP = 22	29.4 ± 0.9	Healthy young men	FiO_2_ 6%			Paced Auditory Serial Addition Test (%)	
Nation et al. (2017) [61]	N = 17 (14M/3F)	30.4 ± 4.7	U.S. Marine Corps and Navy military pilots and aircrews undergoing altitude exposure training	15 min → 6096 m			California Verbal Learning (words)	Wechsler Adult Intelligence Scale (# correct)
Ochi et al. (2018) [46]	N = 29 (20M/9F) EXP 1 = 14 (13M/1F) EXP 2 = 15 (7M/8F)	EXP 1 = 23.4 ± 2.2 EXP 2 = 20.7 ± 2.1	Healthy, dexterous young adults. Native Japanese speakers and naive about experimental procedures.	FiO_2_ 13.5% Washout: non-consecutive sessions	Stroop Test—Neutral (ms) Stroop Test—Incongruent (ms)			
Paul and Fraser (1994) [47]	N = 144	19 to 25	Canadian Forces youths awaiting vocational training, with no experience of decompression at altitude in a hypobaric chamber.	1524 m 2438 m 3048 m 3658 m	Mannikin Task (ms)			
Phillips et al. (2015) [48]	N = 19	Not defined	Active military personnel with a valid flight physical examination	30 min → FiO_2_ 9.96% (5486 m)	Simple RT (ms) Choice RT (ms)			
Piotrowicz et al. (2020) [49]	N = 11	20.0 ± 1.4	Healthy young cyclists	FiO_2_ 14.7% (3000 m) Washout: 5 days	Choice RT (ms)			
Riveros-Rivera et al. (2022) [50]	N = 15 (7M/8F)	29.3 ± 6.6	Healthy people	90 min → FiO_2_ 14.7% 90 min → FiO_2_ 12.5% Washout: 1 week	Stroop Test Incongruent—Congruent (ms)		Digit Span Test (not defined)	Psychomotor Vigilance Test (not defined)
Seo et al. (2015) [51]	N = 16 (M)	24.0 ± 4.0	Young, healthy men	60 min → FiO_2_ 12.5% (4300 m)	Go/No Go Test (ms)	Go/No Go Test (%)	Running Memory Continuous Performance Task (%)	
Seo et al. (2017) [62]	N = 15 (F)	22.0 ± 2.0	Young, healthy women	60 min → FiO_2_ 12.5% (4300 m)			Running Memory Continuous Performance Task (correct response/min)	
Seo (2023) [68]	N = 9 (M)	25.0 ± 2.0	Healthy men, without cardiovascular diseases, metabolic disorders, or respiratory diseases.	30 min → FiO_2_ 17% Washout: 3 days				Stroop Test (interference score)
Smith et al. (2021) [52]	N = 12 (M)	20.9 ± 3.4	Trained persons (3 days/week), non-smokers, without asthma, neuromusculoskeletal disorders, or history of acute mountain sickness.	FiO_2_ 15.4% FiO_2_ 12.9%	Psychomotor vigilance test (ms)			
Thomas et al. (2007) [53]	N = 11 (7M/4F)	27.0 ± 1.5	Healthy people and non-smokers	540 min → FiO_2_ 13% (3962 m)	Psychomotor vigilance test (ms) Verbal 2-back (ms)	Verbal 2-back (%)		
Van Cutsem et al. (2015) [54]	N = 9 (M)	23.0 ± 3.0	Trained athletes	3800 m	Psychomotor vigilance test (ms)			
Wang et al. (2022) [55]	N = 5 (3M/2F)	21.6 ± 0.3	Healthy adults at Army Medical University.	120 min → FiO_2_ 12.8% (4000 m)	Digit Span Task (ms)		Digit Span Test (%)	

Abbreviations: EXP, Experiment; FiO_2_, Fraction of Inspired Oxygen; HYP, Hypoxia Group; NOR, Normoxia/Control Group; RT, Reaction Time; TMT, Trail Making Test.

## Data Availability

All data generated or analyzed during this study are included in this published article.

## Appendix A

- **PUBMED** (18 September 2023)

((((((((((“training” [Title/Abstract]) OR (“exercise” [Title/Abstract])) OR (“program” [Title/Abstract])) OR (“programme” [Title/Abstract])) OR (“intervention” [Title/Abstract])) OR (“proceeding” [Title/Abstract])) OR (“participation” [Title/Abstract])) AND ((((((((“oxygen deficiency” [Title/Abstract]) OR (“deficiencies oxygen” [Title/Abstract])) OR (“hypoxia” [Title/Abstract])) OR (“hypoxemia” [Title/Abstract])) OR (“anoxia” [Title/Abstract])) OR (“anoxemia” [Title/Abstract])) OR (“intermittent hypoxia” [Title/Abstract])) OR (“altitude” [Title/Abstract]))) AND ((((((((((((((“test” [Title/Abstract]) OR (“testing” [Title/Abstract])) OR (“task” [Title/Abstract])) OR (“exam” [Title/Abstract])) OR (“examination” [Title/Abstract])) OR (“battery” [Title/Abstract])) OR (“essay” [Title/Abstract])) OR (“experiment” [Title/Abstract])) OR (“learning” [Title/Abstract])) OR (“measurement” [Title/Abstract])) OR (“work” [Title/Abstract]))) AND ((((((((((((((“cognitive” [Title/Abstract]) OR (“cognitive performance” [Title/Abstract])) OR (“cognitive function” [Title/Abstract])) OR (“psychology” [Title/Abstract])) OR (“neuropsychological” [Title/Abstract])) OR (“neuropsychologic” [Title/Abstract])) OR (“mental” [Title/Abstract])) OR (“psychometric” [Title/Abstract])) OR (“memory” [Title/Abstract])) OR (“reaction time” [Title/Abstract])) OR (“response time” [Title/Abstract])) OR (“anticipation” [Title/Abstract])) OR (“decision making” [Title/Abstract]))
**Results: 340**

- **SCOPUS** (18 September 2023)

(TITLE-ABS (“training”) OR TITLE-ABS (“exercise”) OR TITLE-ABS (“program”) OR TITLE-ABS (“programme”) OR TITLE-ABS (“intervention”) OR TITLE-ABS (“proceeding”) OR TITLE-ABS (“participation”)) AND (TITLE-ABS (“oxygen deficiency”) OR TITLE-ABS (“deficiencies oxygen”) OR TITLE-ABS (“hypoxia”) OR TITLE-ABS (“hypoxemia”) OR TITLE-ABS (“anoxia”) OR TITLE-ABS (“anoxemia”) OR TITLE-ABS (“intermittent hypoxia”) OR TITLE-ABS (“altitude”)) AND (TITLE-ABS (“test”) OR TITLE-ABS (“testing”) OR TITLE-ABS (“task”) OR TITLE-ABS (“exam”) OR TITLE-ABS (“examination”) OR TITLE-ABS (“battery”) OR TITLE-ABS (“essay”) OR TITLE-ABS (“experiment”) OR TITLE-ABS (“learning”) OR TITLE-ABS (“measurement”) OR TITLE-ABS (“work”)) AND (TITLE-ABS (“cognitive”) OR TITLE-ABS (“cognitive performance”) OR TITLE-ABS (“cognitive function”) OR TITLE-ABS (“psychology”) OR TITLE-ABS (“neuropsychological”) OR TITLE-ABS (“neuropsychologic”) OR TITLE-ABS (“mental”) OR TITLE-ABS (“psychometric”) OR TITLE-ABS (“memory”) OR TITLE-ABS (“reaction time”) OR TITLE-ABS (“response time”) OR TITLE-ABS (“anticipation”) OR TITLE-ABS (“decision making”))
**Results: 678**

- **WEB OF SCIENCE** (18 September 2023)

(((AB = (“training” OR “exercise” OR “program” OR “programme” OR “intervention” OR “proceeding” OR “participation”)) AND AB = (“oxygen deficiency” OR “deficiencies oxygen” OR “hypoxia” OR “hypoxemia” OR “anoxia” OR “anoxemia” OR “intermittent hypoxia” OR “altitude”)) AND AB = (“test” OR “testing” OR “task” OR “exam” OR “examination” OR “battery” OR “essay” OR “experiment” OR “learning” OR “measurement” OR “work”)) AND AB = (“cognitive” OR “cognitive performance” OR “cognitive function” OR “psychology” OR “neuropsychological” OR “neuropsychologic” OR “mental” OR “psychometric” OR “memory” OR “reaction time” OR “response time” OR “anticipation” OR “decision making”)
(((TI = (“training” OR “exercise” OR “program” OR “programme” OR “intervention” OR “proceeding” OR “participation”)) AND TI = (“oxygen deficiency” OR “deficiencies oxygen” OR “hypoxia” OR “hypoxemia” OR “anoxia” OR “anoxemia” OR “intermittent hypoxia” OR “altitude”)) AND TI = (“test” OR “testing” OR “task” OR “exam” OR “examination” OR “battery” OR “essay” OR “experiment” OR “learning” OR “measurement” OR “work”)) AND TI = (“cognitive” OR “cognitive performance” OR “cognitive function” OR “psychology” OR “neuropsychological” OR “neuropsychologic” OR “mental” OR “psychometric” OR “memory” OR “reaction time” OR “response time” OR “anticipation” OR “decision making”)
**Results: 408**

- **MEDLINE** (18 September 2023)

(AB “training” OR TI “training” OR AB “exercise” OR TI “exercise” OR AB “program” OR TI “program” OR AB “programme” OR TI “programme” OR AB “intervention” OR TI “intervention” OR AB “proceeding” OR TI “proceeding” OR AB “participation” OR TI “participation”) AND (AB “oxygen deficiency” OR TI “oxygen deficiency” OR AB “deficiencies oxygen” OR TI “deficiencies oxygen” OR AB “hypoxia” OR TI “hypoxia” OR AB “hypoxemia” OR TI “hypoxemia” OR AB “anoxia” OR TI “anoxia” OR AB “anoxemia” OR TI “anoxemia” OR AB “intermittent hypoxia” OR TI “intermittent hypoxia” OR AB “altitude” OR TI “altitude”) AND (AB “test” OR TI “test” OR AB “testing” OR TI “testing” OR AB “task” OR TI “task” OR AB “exam” OR TI “exam” OR AB “examination” OR TI “examination” OR AB “battery” OR TI “battery” OR AB “essay” OR TI “essay” OR AB “experiment” OR TI “experiment” OR AB “learning” OR TI “learning” OR AB “measurement” OR TI “measurement” OR AB “work” OR TI “work”) AND (AB “cognitive” OR TI “cognitive” OR AB “cognitive performance” OR TI “cognitive performance” OR AB “cognitive function” OR TI “cognitive function” OR AB “psychology” OR TI “psychology” OR AB “neuropsychological” OR TI “neuropsychological” OR AB “neuropsychologic” OR TI “neuropsychologic” OR AB “mental” OR TI “mental” OR AB “psychometric” OR TI “psychometric” OR AB “memory” OR TI “memory” OR AB “reaction time” OR TI “reaction time” OR AB “response time” OR TI “response time” OR AB “anticipation” OR TI “anticipation” OR AB “decision making” OR TI “decision making”)
**Results: 309**

- **SPORTDISCUS** (18 September 2023)

(AB “training” OR TI “training” OR AB “exercise” OR TI “exercise” OR AB “program” OR TI “program” OR AB “programme” OR TI “programme” OR AB “intervention” OR TI “intervention” OR AB “proceeding” OR TI “proceeding” OR AB “participation” OR TI “participation”) AND (AB “oxygen deficiency” OR TI “oxygen deficiency” OR AB “deficiencies oxygen” OR TI “deficiencies oxygen” OR AB “hypoxia” OR TI “hypoxia” OR AB “hypoxemia” OR TI “hypoxemia” OR AB “anoxia” OR TI “anoxia” OR AB “anoxemia” OR TI “anoxemia” OR AB “intermittent hypoxia” OR TI “intermittent hypoxia” OR AB “altitude” OR TI “altitude”) AND (AB “test” OR TI “test” OR AB “testing” OR TI “testing” OR AB “task” OR TI “task” OR AB “exam” OR TI “exam” OR AB “examination” OR TI “examination” OR AB “battery” OR TI “battery” OR AB “essay” OR TI “essay” OR AB “experiment” OR TI “experiment” OR AB “learning” OR TI “learning” OR AB “measurement” OR TI “measurement” OR AB “work” OR TI “work”) AND (AB “cognitive” OR TI “cognitive” OR AB “cognitive performance” OR TI “cognitive performance” OR AB “cognitive function” OR TI “cognitive function” OR AB “psychology” OR TI “psychology” OR AB “neuropsychological” OR TI “neuropsychological” OR AB “neuropsychologic” OR TI “neuropsychologic” OR AB “mental” OR TI “mental” OR AB “psychometric” OR TI “psychometric” OR AB “memory” OR TI “memory” OR AB “reaction time” OR TI “reaction time” OR AB “response time” OR TI “response time” OR AB “anticipation” OR TI “anticipation” OR AB “decision making” OR TI “decision making”)

## Appendix B

**Table A1:** PEDro scale.

Study	Criterion 1	Criterion 2	Criterion 3	Criterion 4	Criterion 5	Criterion 6	Criterion 7	Criterion 8	Criterion 9	Criterion 10	Criterion 11	Total
Ando et al. (2010) [34]	0	1	1	0	1	0	0	1	1	1	1	7
Ando et al. (2013) [33]	0	1	1	0	1	0	0	1	1	1	1	7
Chroboczek et al. (2021) [63]	1	0	0	1	1	0	0	1	1	1	1	6
Chroboczek et al. (2022) [64]	0	0	0	0	1	0	0	1	1	1	1	5
Dobashi et al. (2016) [56]	0	0	0	0	0	0	0	1	1	1	1	4
Feeback et al. (2017) [65]	1	0	0	0	0	0	0	1	1	1	1	4
Fowler et al. (1985) [35]	0	1	0	0	1	0	0	1	1	1	1	6
Guicciardi et al. (2022) [36]	1	1	1	1	0	0	0	1	1	1	1	7
Heled et al. (2012) [37]	0	0	0	0	0	0	0	1	1	1	1	4
Hohenauer et al. (2022) [38]	1	1	1	0	1	0	0	1	1	1	1	7
Jenkins et al. (2022) [57]	0	1	0	0	1	0	0	1	1	1	1	6
Jimenez et al. (2019) [39]	1	1	1	0	1	0	0	1	1	1	1	7
Karayigit et al. (2022) [40]	1	1	1	0	1	1	1	1	1	1	1	9
Kim et al. (2015) [66]	0	0	0	0	0	0	0	1	1	1	1	4
Komiyama et al. (2015) [58]	0	1	1	1	1	0	0	1	1	1	1	8
Komiyama et al. (2017) [10]	0	1	1	0	1	0	0	1	1	1	1	7
Kong et al. (2022) [41]	0	1	1	0	1	0	0	1	1	1	1	7
Ledwith (1970) [42]	0	1	0	0	1	0	0	1	1	1	1	6
Lefferts et al. (2016) [43]	1	1	0	1	0	0	0	1	1	1	1	6
Lei et al. (2019) [44]	1	1	1	0	1	0	0	1	1	1	1	7
Lei et al. (2022) [45]	1	1	1	1	1	0	0	1	1	1	1	8
Limmer and Platen (2018) [67]	0	0	0	1	0	0	0	1	1	1	1	5
Malle et al. (2013) [60]	0	1	0	1	0	0	0	1	1	1	1	6
Malle et al. (2016) [59]	0	1	1	1	1	0	0	1	1	1	1	8
Nation et al. (2017) [61]	0	0	0	1	0	0	0	1	1	1	1	5
Ochi et al. (2018) [46]	0	1	0	0	0	0	0	1	1	1	1	5
Paul and Fraser (1994) [47]	0	1	1	0	1	0	0	1	1	1	1	7
Phillips et al. (2015) [48]	0	0	0	1	0	0	0	1	1	1	1	5
Piotrowicz et al. (2020) [49]	0	1	1	0	0	0	0	1	1	1	1	6
Riveros-Rivera et al. (2022) [50]	1	1	1	0	1	0	0	1	1	1	1	7
Seo et al. (2015) [51]	1	0	0	1	0	0	0	1	1	1	1	5
Seo et al. (2017) [62]	1	0	0	1	0	0	0	1	1	1	1	5
Seo (2023) [68]	0	0	1	0	1	0	0	1	1	1	1	6
Smith et al. (2021) [52]	0	1	0	0	1	0	0	1	1	1	1	6
Thomas et al. (2007) [53]	0	0	0	0	0	0	0	1	1	1	1	4
Van Cutsem et al. (2015) [54]	0	1	1	0	1	1	1	1	1	1	1	9
Wang et al. (2022) [55]	0	0	0	0	0	0	0	1	1	1	1	4

Criterion 1 eligibility criteria were specified; Criterion 2 subjects were randomly allocated to groups; Criterion 3 allocation was concealed; Criterion 4 groups were similar at baseline regarding the most important prognostic indicators; in Criterion 5, there was blinding of all subjects; in Criterion 6, there was blinding of all therapists who administered the therapy; in Criterion 7, there was blinding of all assessors who measured at least one key outcome; Criterion 8 measures of at least one key outcome were obtained from more than 85% of the subjects initially allocated to groups; in Criterion 9, all subjects for whom outcome measures were available received the treatment or control condition as allocated; in Criterion 10, the results of between-group statistical comparisons are reported for at least one key outcome; in Criterion 11, the study provides both point measures and measures of variability for at least one key outcome.

## Appendix C

**Table A2:** Risk of bias assessment of the included trials.

Study	Risk of Bias
Ando et al. (2010) [34]	Some concerns
Ando et al. (2013) [33]	Some concerns
Chroboczek et al. (2021) [63]	Some concerns
Chroboczek et al. (2022) [64]	Some concerns
Dobashi et al. (2016) [56]	Some concerns
Feeback et al. (2017) [65]	Some concerns
Fowler et al. (1985) [35]	Some concerns
Guicciardi et al. (2022) [36]	Some concerns
Heled et al. (2012) [37]	Some concerns
Hohenauer et al. (2022) [38]	Some concerns
Jenkins et al. (2022) [57]	Some concerns
Jimenez et al. (2019) [39]	Low risk of bias
Karayigit et al. (2022) [40]	Low risk of bias
Kim et al. (2015) [66]	Some concerns
Komiyama et al. (2015) [58]	Low risk of bias
Komiyama et al. (2017) [10]	Some concerns
Kong et al. (2022) [41]	Low risk of bias
Ledwith (1970) [42]	Some concerns
Lefferts et al. (2016) [43]	Some concerns
Lei et al. (2019) [44]	Some concerns
Lei et al. (2022) [45]	Some concerns
Limmer and Platen (2018) [67]	Some concerns
Malle et al. (2013) [60]	Some concerns
Malle et al. (2016) [59]	Low risk of bias
Nation et al. (2017) [61]	Some concerns
Ochi et al. (2018) [46]	Some concerns
Paul and Fraser (1994) [47]	Some concerns
Phillips et al. (2015) [48]	Some concerns
Piotrowicz et al. (2020) [49]	Some concerns
Riveros-Rivera et al. (2022) [50]	Low risk of bias
Seo et al. (2015) [51]	Some concerns
Seo et al. (2017) [62]	Some concerns
Seo (2023) [68]	Some concerns
Smith et al. (2021) [52]	Low risk of bias
Thomas et al. (2007) [53]	Some concerns
Van Cutsem et al. (2015) [54]	Low risk of bias
Wang et al. (2022) [55]	Some concerns

Note: If a study’s scores are “+” in all subdomains, the overall rating is “low risk of bias”. When a study’s scores are “?” in one or more subdomains, the overall rating is considered “some concerns”. If a study’s scores are “-” in one or more subdomains, the overall rating is “high risk of bias”, giving rise to substantial doubts about the quality of the research.
